# Analysis of clinical characteristics, pathogen infection, and drug sensitivity of Marine injury patients: A cross-sectional study

**DOI:** 10.1097/MD.0000000000029943

**Published:** 2022-07-29

**Authors:** Lei Ge, Yang Gao, Kai Wang, Qiandong Liu, Panpan Cui, Qinglin Dong

**Affiliations:** a Department of Emergency, People’s Hospital of Rizhao, Jining Medical University, Shandong, China; b Department of Anesthesiology, People’s Hospital of Rizhao, Jining Medical University, Shandong, China; c Department of Otorhinolaryngology, People’s Hospital of Rizhao, Jining Medical University, Shandong, China.

**Keywords:** bacterial, drug tolerance, naval medicine

## Abstract

The infection rate is high in patients injured at sea, and because of the unique distribution of marine microorganisms, the infection is often not easily controlled effectively with the empirical application of antibiotics. This study aims to consider the clinical characteristics and pathogen infection and drug susceptibility of patients injured at sea. From 2019 to 2021, there were 635 patients injured at sea in Rizhao People’s Hospital. We assess the patient’s basic condition, while performing bacterial culture and drug susceptibility testing on wound exudate or pus from infected patients. Among the 635 patients injured at sea, 195 people were infected, and the infection rate was 30.71%. Infected patients are usually older, have longer prehospital visits, and have lower normal levels of red blood cells, hemoglobin, total protein, and albumin. The causes of injury in infected patients were mainly avulsion and puncture injuries, and the types of injuries were mainly bone fracture, vascular injury, and nerve injury. A total of 305 strains of pathogenic bacteria were cultured in 195 patients. Gram-negative bacteria accounted for 77.05% (235 strains), of which Proteus was the most. Gram-positive bacteria accounted for 22.95% (70 strains), of which *Staphylococcus aureus* was the most. Gram-negative bacilli were sensitive to aminoglycosides, lactam antibiotics, carbapenems antibiotics, sulfonamides, quinolones, fourth-generation cephalosporins, and antibacterial drugs containing enzyme inhibitors, while most of the bacteria were resistant to penicillins, first-generation cephalosporins, and second-generation cephalosporins. Gram-positive bacteria were sensitive to quinuptin/dafoptin, rifampicin, linezolid, gentamicin, tigacycline, and vancomycin but resistant to penicillin antibiotics. Due to the particularity of marine injuries, patients are prone to infection. Pathogen culture and drug sensitivity analysis play an important role in guiding antiinfective treatment for marine injured patients.

## 1. Introduction

Now with the development of the marine economy and the increase in maritime activities such as maritime military activities, maritime accidents are frequent and more and more patients are injured at sea. For patients injured at sea, the wounds are easily soaked by sea water. Unlike terrestrial damage, seawater has very specific physicochemical properties such as low temperature, hypertonicity, high sodium, high chloride, slightly alkaline, and high bacterial content.

There are many types and numbers of bacteria in seawater.^[1]^ In our previous study of seawater from China’s seas, we found that Vibrio was the most common, followed by Enterobacteriaceae and nonfermenting bacteria. At the same time, *Staphylococcus aureus, Staphylococcus hemolyticus*, balloon bacteria, *Clostridium innocuous and Eubacterium mucilage* were also isolated.

The treatment of marine injured patients has always been the research focus of coastal medical institutions in my country. In clinical work, surgical treatment and broad-spectrum antibiotics are routinely used to prevent and control infection in patients with marine injury, but it was found that the infection rate of patients with marine injury was significantly higher than that of other trauma patients.^[2]^

Rizhao People’s Hospital is a regional hospital. It is the largest general hospital in the coastal area of Shandong, China. It is also a national marine bacteria monitoring outpost. These cases cover the city of Rizhao and the surrounding areas, which is of great significance for the study of the treatment of marine injury and infection.

## 2. Objectives

This study investigated the epidemiological characteristics of patients with marine injuries, especially in age, month distribution, injury mechanism, prehospital visit time, hospitalization time, injury type, blood pressure, blood glucose, serological indexes, bacterial culture, and drug sensitivity, to provide the basis for the antiinfection treatment of patients with marine injuries.

## 3. Methods

### 3.1. Study subjects

A total of 635 orthopedic patients admitted to our hospital for marine injuries from March 2019 to March 2021 were retrospectively selected. Inclusion criteria: 1. Clearly injured patients at sea 2. Complete clinical case records; 3. There are 2 or more bacterial culture and drug susceptibility test results; 4. Complete imaging records. Exclusion criteria: 1. No injuries at sea 2. preinjury infection; 3. Incomplete clinical case records; 3. There is no clear bacterial culture and drug susceptibility test results, or bacterial culture results cannot rule out contamination; 4. No imaging data. The study was done after agreement from the local ethics committee and with the patients’ informed consent.

### 3.2. Instruments and reagents

VITEK 2 compact automated microbial identification system and biological merrier API identification system. Drug susceptibility paper, Mueller Hinton dry powder, and Haemophilus test dry powder were purchased from British Oxoid Company.

### 3.3. Bacterial culture

After scrubbing the surface of the lesion with sterile saline, the pus and secretions deep inside the lesion were collected by a trained and experienced physician with a sterile swab moistened with sterile saline, and sent immediately to the microbiology laboratory.

### 3.4. Bacterial identification and antimicrobial susceptibility test

Bacteria were identified by VITEKT2 Compact System (USA). The Minimum Inhibitory concentration (MIC) of noncaustic bacteria and streptococci was determined by VitekT2 Compact System. The MIC of other caustic bacteria was determined by the Kirby Bauer paper diffusion method. Drug sensitivity criteria follow the criteria of the American Institute of Clinical and Laboratory Standards (CLSI).

### 3.5. Statistical analysis

SPSS software was used to analyze these data. Data description was expressed as mean ± standard deviation (x ± s), *T*-test was used for pairwise comparisons, and ANOVA test was used for multiple variables. Test *P* < .05 indicates statistical significance.

## 4. Results

### 4.1. Comparison of overall conditions between infected and uninfected patients

**Figure 1.**
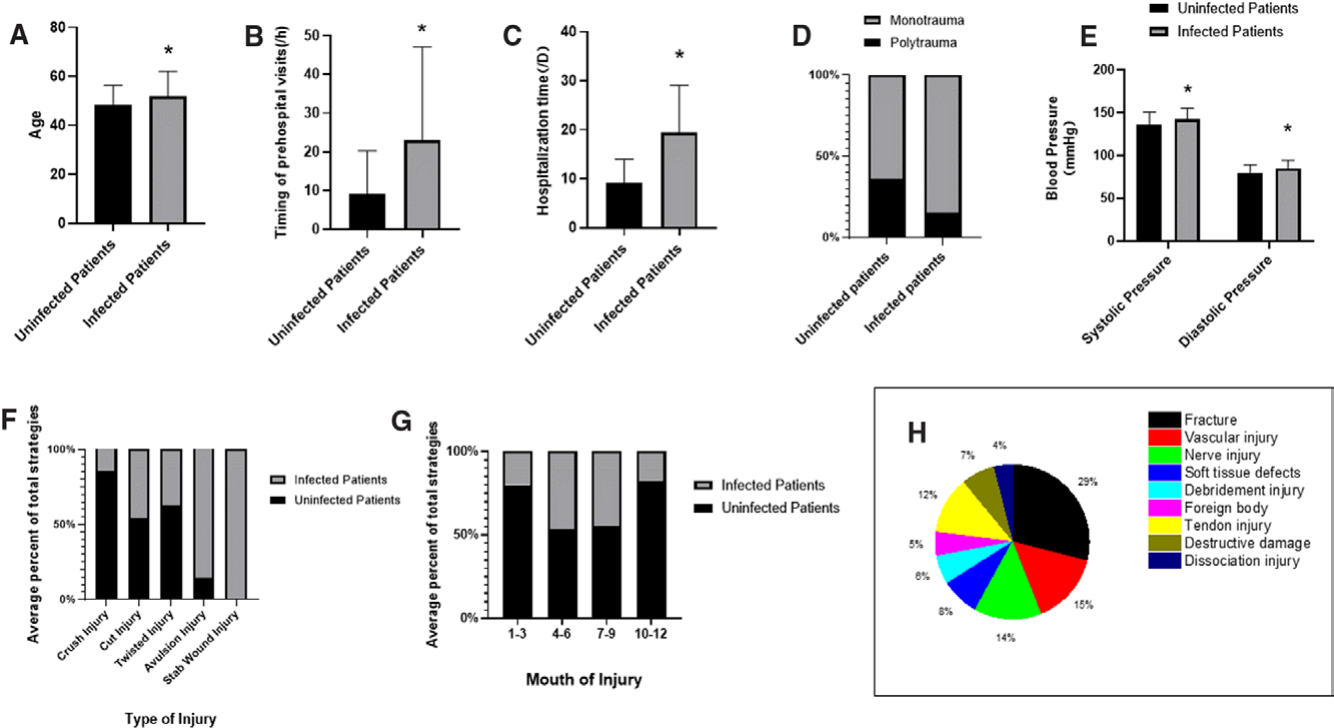
Comparison of overall conditions between infected and uninfected patients The age, prehospital visit time, hospitalization time, and blood pressure of infected patients were significantly higher than those of uninfected patients. Infected patients are most common from April to September, and the main types are avulsion injury and stab wound injury, and the main types of injuries were fracture, vascular injury, and nerve injury. *P < .05 vs uninfected patients.

A total of 635 cases were collected, including 579 males and 56 females, of which 195 were infected, with a contact rate of 30.71%. There were 195 infected patients, 170 males and 25 females, aged 51.89 ± 10.07 years. The prehospital visit time was 23.08 ± 23.98 hours, and the hospitalization time was 19.46 ± 9.66 days. There were 440 infected cases, 409 males and 31 females, aged 48.31 ± 8.02 years. The prehospital visit time was 9.11 ± 11.18 hours and the hospitalization time was 9.16 ± 4.79 days. The infection probability was 29.36% in male patients and 44.64% in female patients. The age, prehospital visit time, and hospitalization time were significantly higher in infected patients than in uninfected patients (Fig. 1A–C). In terms of injury mechanism, the infection rate of avulsion and stab wounds was significantly over 50% (Fig. 1F). The infection rate of patients from April to September was significantly higher than other months (Fig. 1G). The proportion of multiple injuries in patients with marine injury and infection was significantly higher than that in patients without infection (Fig. 1D), and the main injury types were fractures, vascular injury, and nerve injury (Fig. 1H). Infected patients had higher systolic and diastolic blood pressures than uninfected patients (Fig. 1E). The above differences are statistically significant.

### 4.2. Comparison of serological indexes between infected patients and uninfected patients

**Figure 2.**
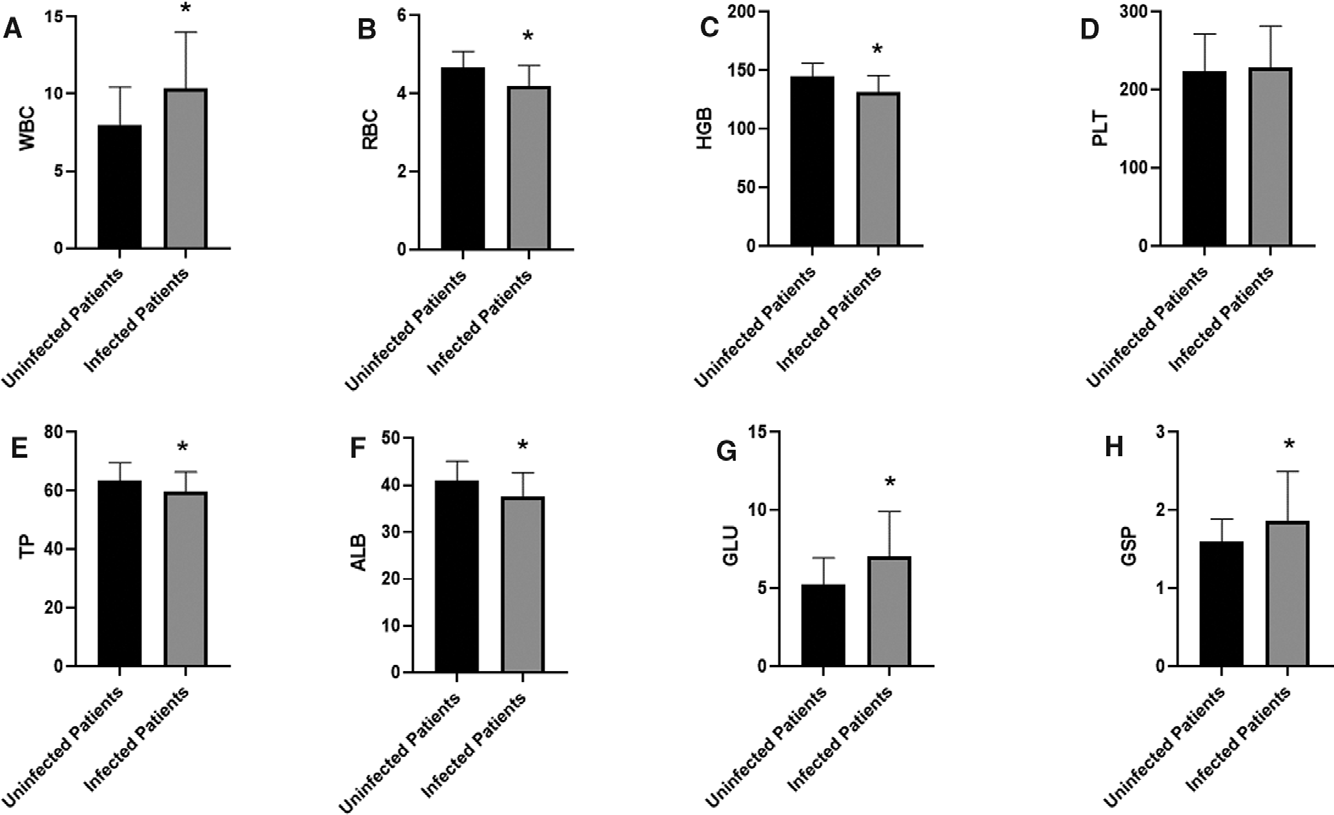
Comparison of serological indexes between infected patients and uninfected patients. The WBC, GLU, and GSP of the infected patients were significantly higher than those of the uninfected patients, and the RBC, HGB, TP, and ALB of the infected patients were significantly lower than those of the uninfected patients. *P < .05 vs uninfected patients.

By analyzing the white blood cells, red blood cells, hemoglobin, platelets, total protein, albumin, glucose, and glycosylated serum protein of all patients, we found that the WBC, GLU, and GSP of infected patients were significantly higher than those of uninfected patients (Fig. 2A,G,H). The RBC, HGB, TP, and ALB of infected patients were significantly lower than those of uninfected patients (Fig. 2B–F). There were no significant differences in PLT (Fig. 2D). The above differences are statistically significant.

### 4.3. Bacterial species and drug sensitivity analysis of marine injury infection patients

305 strains of pathogenic bacteria were co-cultured in 195 patients. Gram-negative bacteria were 235 strains (77.0%), including 45 strains of Proteus bacteria (14.8%), and the rest were Shewanella algae, *Aeromonas hydrophila, Enterobacter cloacae* (Table 1). Gram-positive bacteria accounted for 70 (23.0%), *Staphylococcus aureus* accounted for 17.4% (53 strains), and the rest were *Enterococcus faecalis* and *Streptococcus viridans* (Table 1).

**Table 1:** Bacterial distribution in 195 patients with marine injury and infection in Rizhao, China, within 2019–2021.

Pathogen species	Name	Number of bacteria	Constituent ratio %
Gram-negative bacteria		235	77.0
	Shewanella algae	31	10.2
	*Vibrio alginolyticus*	9	3.0
	*Citrobacter braakii*	10	3.3
	*Klebsiella oxytoca*	7	2.3
	*Morganella morganii*	18	5.9
	*Photobacterium damselae*	5	1.6
	Proteus bacteria	45	14.8
	*Enterobacter aerogenes*	4	1.3
	*Pseudomonas aeruginosa*	21	6.9
	*Aeromonas hydrophila*	25	8.2
	*Serratia marcescens*	9	3.0
	*Pseudomonas stutzeri*	9	3.0
	*Enterobacter cloacae*	27	8.9
	*Vibrio vulnificus*	15	4.9
Gram-positive		70	23.0
	*Staphylococcus aureus*	53	17.4
	*Enterococcus faecalis*	11	3.6
	*Viridans Streptococci*	6	2.0
total		305	100.0

Gram-negative bacilli were sensitive to aminoglycosides, lactam antibiotics, carbapenems antibiotics, sulfonamides, quinolones, fourth-generation cephalosporins, and antibacterial drugs containing enzyme inhibitors, while most of the bacteria were resistant to penicillins, first-generation cephalosporins, and second-generation cephalosporins (Tables 2 and 3). gram-positive bacteria, are sensitive to quinidine/ dafutin, rifampicin, linezolid, gentamicin, tegacyclin and vancomycin, but resistant to penicillin antibiotics (Tables 4 and 5).

**Table 2:** Resistance rate of main Gram-negative bacteria to common antibiotics (%).

	Shewanella algae	*Morganella morganii*	Proteus bacteria	*Pseudomonas aeruginosa*	*Aeromonas hydrophila*	*Enterobacter cloacae*
Ampicillin	0.0	0.0	55.6	0.0	60.0	0.0
Ampicillin sulbactam	16.7	75.0	55.6	0.0	80.0	0.0
Aztreonam	33.3	0.0	0.0	0.0	20.0	0.0
Ertapenem	0.0	0.0	11.1	0.0	20.0	0.0
Compound sulfamethoxazole	16.7	25.0	33.3	0.0	60.0	20.0
Ciprofloxacin	33.3	25.0	33.3	0.0	40.0	0.0
Piperacillin	16.7	25.0	22.2	0.0	0.0	20.0
Piperacillin tazobactam	16.7	0.0	0.0	0.0	20.0	0.0
Gentamicin	16.7	25.0	33.3	0.0	0.0	0.0
Cefepime	16.7	0.0	22.2	0.0	20.0	0.0
Cefuroxime	16.7	25.0	44.4	0.0	0.0	0.0
Cefoperazone sulbactam	16.7	0.0	0.0	25.0	0.0	0.0
Cefatriaxone	16.7	0.0	22.2	0.0	40.0	0.0
Ceftazidime	16.7	0.0	11.1	0.0	20.0	0.0
Cefoxitin	16.7	0.0	0.0	0.0	20.0	0.0
Cefazolin	33.3	25.0	100.0	50.0	80.0	60.0
Tobramycin	0.0	25.0	22.2	0.0	20.0	0.0
Imipenem	33.3	0.0	11.1	50.0	20.0	0.0
Levofloxacin	33.3	25.0	0.0	0.0	20.0	0.0

**Table 3:** Sensitivity rate of main Gram-negative bacteria to common antibiotics (%).

	Shewanella algae	*Morganella morganii*	Proteus bacteria	*Pseudomonas aeruginosa*	*Aeromonas hydrophila*	*Enterobacter cloacae*
Amikacin	100.0	100.0	88.9	100.0	80.0	100.0
Ampicillin sulbactam	0.0	25.0	33.3	0.0	0.0	0.0
Aztreonam	50.0	100.0	88.9	50.0	80.0	100.0
Ertapenem	0.0	100.0	77.8	0.0	20.0	100.0
Compound Sulfamethoxazole	16.7	75.0	55.6	0.0	40.0	80.0
Ciprofloxacin	16.7	75.0	55.6	100.0	60.0	100.0
Meropenem	66.7	100.0	77.8	25.0	20.0	60.0
Minocycline	50.0	100.0	77.8	0.0	20.0	60.0
Piperacillin	50.0	75.0	55.6	25.0	20.0	40.0
Piperacillin tazobactam	83.3	100.0	88.9	100.0	80.0	100.0
Gentamicin	50.0	75.0	55.6	100.0	100.0	100.0
Tigecycline	16.7	50.0	55.6	0.0	40.0	80.0
Cefepime	83.3	100.0	77.8	100.0	80.0	100.0
Cefuroxime	0.0	25.0	22.2	0.0	20.0	40.0
Cefoperazone sulbactam	66.7	100.0	77.8	0.0	20.0	60.0
Cefatriaxone	16.7	100.0	66.7	0.0	60.0	100.0
Cefotaxime	0.0	100.0	55.6	0.0	20.0	60.0
Ceftazidime	83.3	100.0	77.8	100.0	60.0	80.0
Cefotetan	0.0	100.0	88.9	0.0	40.0	0.0
Cefazolin	0.0	25.0	0.0	0.0	20.0	0.0
Tobramycin	16.7	75.0	66.7	100.0	40.0	100.0
Imipenem	66.7	0.0	66.7	50.0	80.0	100.0
Levofloxacin	50.0	75.0	88.9	100.0	80.0	100.0

**Table 4:** Resistance rate of main Gram-positive bacteria to common antibiotics (%).

	*Staphylococcus aureus*	*Enterococcus faecalis*	*Viridans Streptococci*
Ampicillin	0.0	100.0	0.0
Oxacillin	72.7	0.0	0.0
Compound sulfamethoxazole	90.9	0.0	0.0
Erythromycin	45.5	50.0	0.0
Ciprofloxacin	72.7	50.0	0.0
Clindamycin	45.5	0.0	0.0
Quinuptin/dafoptin	100.0	0.0	0.0
Rifampicin	100.0	0.0	0.0
Linezolid	100.0	100.0	100.0
Streptomycin	0.0	100.0	0.0
Moxifloxacin	72.7	0.0	0.0
Penicillin	9.1	100.0	100.0
Gentamicin	100.0	100.0	0.0
Tetracyclines	81.8	100.0	0.0
Tigecycline	100.0	100.0	0.0
Cefepime	0.0	0.0	100.0
Ceftriaxone	0.0	0.0	100.0
Vancomycin	100.0	100.0	100.0
Levofloxacin	72.7	100.0	100.0

**Table 5:** Sensitivity rate of main Gram-positive bacteria to common antibiotics (%).

	*Staphylococcus aureus*	*Enterococcus faecalis*	*Viridans Streptococci*
Ampicillin	0.0	100.0	0.0
Oxacillin	72.7	0.0	0.0
Compound Sulfamethoxazole	90.9	0.0	0.0
Erythromycin	45.5	50.0	0.0
Ciprofloxacin	72.7	50.0	0.0
Clindamycin	45.5	0.0	0.0
Quinuptin/dafoptin	100.0	0.0	0.0
Rifampicin	100.0	0.0	0.0
Linezolid	100.0	100.0	100.0
Streptomycin	0.0	100.0	0.0
Moxifloxacin	72.7	0.0	0.0
Penicillin	9.1	100.0	100.0
Gentamicin	100.0	100.0	0.0
Tetracyclines	81.8	100.0	0.0
Tigecycline	100.0	100.0	0.0
Cefepime	0.0	0.0	100.0
Ceftriaxone	0.0	0.0	100.0
Vancomycin	100.0	100.0	100.0
Levofloxacin	72.7	100.0	100.0

## 5. Discussion

There were 579 males in 635 cases, accounting for 91.18%. This may be related to the predominance of male workers in marine jobs. The probability of infection in male patients was 29.36%, and the probability of infection in female patients was 44.64%. Female patients have a higher chance of infection, which may be related to the poorer physical fitness of women. The present study showed an infection rate of 30.71% in patients injured at sea, which is consistent with previous studies.^[3]^ Patients with infection generally present with advanced age, hypertension, anemia, low protein, and hyperglycemia, and this result indicates a correlation between the occurrence of infection and the patient’s health.^[4]^ At the same time, we found that the prehospital visit time of infected patients is long, far exceeding the optimal treatment time, which may be an important factor leading to infection, and the long prehospital visit time can also lead to a large amount of blood loss. These are an important reason for the high white blood cell count and low red blood cell count in infected patients. Therefore, the length of prehospital visit time may be directly related to the occurrence of infection in patients.

The infection of injured personnel at sea has obvious seasonality, and it is mostly concentrated in the second half of the year, which is related to the large amount of offshore operations and bad weather. The main cause of injury is crush injury, fracture is the most common type of injury, which is related to the nature of work.

Infections caused by injuries at sea have obvious seasonality and are more concentrated in the second half of the year, which is related to heavy offshore operations and bad weather. Because the offshore operation site is far away from the land, and the transportation capacity of marine patients is weak, it often takes a long time for patients to arrive at the hospital after being injured. This is also a very important cause of infection.

Among the 305 strains of pathogenic bacteria, 235 strains were Gram-negative bacteria, accounting for 77.05%, and 70 strains were Gram-positive bacteria, accounting for 22.95%. Gram-negative bacteria include Proteus bacteria, Shivarella alga, Aeromonas hydrophila, and Enterobacter cloacae, among which there are special Marine bacteria, such as Vibrio vulnificus, Vibrio alginolyticus, and Photobacterium damselae. The distribution of pathogenic bacteria is different from that of bacteria infected with trauma.^[5,6]^ It can be seen from the drug sensitivity that Gram-negative are sensitive to aminoglycosides, lactam antibiotics, carbapenems antibiotics sulfonamides quinolones, fourth-generation cephalosporins, and antibacterial drugs containing enzyme inhibitors, while most bacteria are resistant to first-generation cephalosporins and second-generation penicillin antibiotics. Multiple resistance mechanisms have been demonstrated, including antibiotic degradation, antibiotic target modification, and regulation of permeability through bacterial membranes.^[7]^

There were 15 cases of Vibrio vulnificus infection in this group. Vibrio vulnificus infection has been associated with eating contaminated seafood, open wounds exposed to seawater, and gastrointestinal infections. The mortality rate of Vibrio vulnificus infection is higher than 50%. Treatment of Vibrio vulnificus is prompt administration of high doses of sensitive antibiotics and early surgical intervention.^[8]^ The treatment of patients with Vibrio vulnificus sepsis complicated with cellulitis or necrotizing fasciitis should not only require early surgical treatment, but also actively choose decompression and debridement surgery, and amputation if necessary.^[9]^

gram-positive bacteria, most of which are *Staphylococcus aureus*, are sensitive to quinidine/dafutin, rifampicin, linezolid, gentamicin, tegacyclin and vancomycin, but resistant to penicillin antibiotics. The resistance mechanism is associated with high levels of β-lactam resistance in *Staphylococcus aureus*, which is associated with alterations in RNA polymerase and fine-tuning of gene expression.^[10]^

In conclusion, Due to the particularity of marine injuries, patients are prone to infection. It is necessary to strengthen the training of medical personnel on board, and timely perform simple disinfection and hemostatic bandaging on injured patients, which can effectively reduce the chance of wound exposure. In addition, it is necessary to strengthen the transportation force of patients, especially the maritime air ambulance transportation force, and shorten the prehospital treatment time, which is also one of the important measures to reduce the infection of marine trauma. After the patient is admitted to the hospital, it is necessary to actively and thoroughly handle the wound, apply broad-spectrum effective antibiotics in early and sufficient quantity, closely observe the changes of the whole body and wound conditions, conduct bacterial culture and drug sensitivity test in early stage, and adjust the use of antibiotics at any time according to the results of drug sensitivity test.

## Author contributions

Conceptualization: Lei Ge, Yang Gao

Data curation: Lei Ge, Kai Wang, Panpan Cui

Formal analysis: Lei Ge

Investigation: Yang Gao

Resources: Qiandong Liu, Qinglin Dong

Software: Kai Wang

Writing – original draft: Lei Ge, Panpan Cui

Writing – review & editing: Yang Gao.
